# Plant biostimulants as natural alternatives to synthetic auxins in strawberry production: physiological and metabolic insights

**DOI:** 10.3389/fpls.2023.1337926

**Published:** 2024-01-09

**Authors:** Mariateresa Cardarelli, Antonio El Chami, Youssef Rouphael, Michele Ciriello, Paolo Bonini, Gorka Erice, Veronica Cirino, Boris Basile, Giandomenico Corrado, Seunghyun Choi, Hye-Ji Kim, Giuseppe Colla

**Affiliations:** ^1^ Department of Agriculture and Forest Sciences, University of Tuscia, Viterbo, Italy; ^2^ Department of Agricultural Sciences, University of Naples Federico II, Portici, Italy; ^3^ OloBion SL, Barcelona, Spain; ^4^ Atens - Agrotecnologías Naturales, La Riera de Gaià, Spain; ^5^ Texas A&M AgriLife Research and Extension Center, Texas A&M University, Uvalde, TX, United States; ^6^ Agri-tech and Food Innovation Department, Urban Food Solutions Division, Singapore Food Agency, Singapore, Singapore

**Keywords:** *Fragaria*, bacterial filtrate, protein hydrolysate, naphthaleneacetamide, naphthaleneacetic acid, fruit yield, fruit quality, metabolomics

## Abstract

The demand for high-quality strawberries continues to grow, emphasizing the need for innovative agricultural practices to enhance both yield and fruit quality. In this context, the utilization of natural products, such as biostimulants, has emerged as a promising avenue for improving strawberry production while aligning with sustainable and eco-friendly agricultural approaches. This study explores the influence of a bacterial filtrate (BF), a vegetal-derived protein hydrolysate (PH), and a standard synthetic auxin (SA) on strawberry, investigating their effects on yield, fruit quality, mineral composition and metabolomics of leaves and fruits. Agronomic trial revealed that SA and BF significantly enhanced early fruit yield due to their positive influence on flowering and fruit set, while PH treatment favored a gradual and prolonged fruit set, associated with an increased shoot biomass and sustained production. Fruit quality analysis showed that PH-treated fruits exhibited an increase of firmness and soluble solids content, whereas SA-treated fruits displayed lower firmness and soluble solids content. The ionomic analysis of leaves and fruits indicated that all treatments provided sufficient nutrients, with heavy metals within regulatory limits. Metabolomics indicated that PH stimulated primary metabolites, while SA and BF directly affected flavonoid and anthocyanin biosynthesis, and PH increased fruit quality through enhanced production of beneficial metabolites. This research offers valuable insights for optimizing strawberry production and fruit quality by harnessing the potential of natural biostimulants as viable alternative to synthetic compounds.

## 1 Introduction

One of the biggest issues facing the agriculture sector is feeding an expanding global population while minimizing its environmental impact and protecting natural resources for future generations (Devaux et al., 2021). In this context, the Food and Agriculture Organization of the United Nations (FAO) has set a vision about sustainable agriculture which is based on preservation of the natural resources and a technical transformation that is focused on ensuring the fulfilment of continual human requirements for both current and future generations (FAO, 2019). In addition, the FAO has set a strategic objective for sustainable intensification of crop production and focuses on switching to alternative intensification methods, depending on biodiversity management and natural biological processes to boost agroecosystem output while dealing with issues related to climate change and having a good influence on the environment (FAO, 2019). Synthetic hormones (e.g., napthaleneacetic acid 6-benzyladenine (NAA), gibberellins, cytokinins, abscisic acid (ABA), ethylene, brassinosteroids, and jasmonates), the so-called plant growth regulators (PGRs), are largely used in horticulture to enhance the production of vegetables and fruits (Dias, 2019). Moreover, the control mechanisms of phytohormones in fruit set have drawn a lot of attention in recent years because these technologies have been used in horticulture and agriculture to produce seedless fruits and boost crop productivity and quality. These advancements prove beneficial not only in optimal growing conditions but also in challenging and unfavorable environments (e.g., short growing seasons, non-suitable environment for fertilization, low soil fertility, and diseases) (Taglienti et al., 2011; Cho et al., 2013; Farman et al., 2019; Sharif et al., 2022). For instance, several studies elucidated the effect of synthetic auxin (NAA) and gibberellins (GAs) application on enhancing fruit set and growth (McAtee et al., 2013; Kumar et al., 2014) and on enhancing fruit size and quality in many fruits such as plum (Stern et al., 2006), apple (Devoghalaere et al., 2012), apricot (Stern et al., 2007), loquat (Amorós et al., 2004; Forlani et al., 2010), tomato (Zhang et al., 2021), strawberry (Thakur et al., 2017). On the other hand, several studies have shown that the residues of PGRs applied in agricultural crops, have been linked to genotoxicity, hepatotoxicity, and renal toxicity, all of which pose substantial risks to human health (Lin and Tan, 2011; Maslowski and Mackay, 2011; Magnone et al., 2015; Reihill et al., 2015). Being the PGRs registered as plant protection products, where rates, application methods, and safety intervals for each use between application and harvest have been defined and approved by international and national authorities to control PGR residues in crops as well as to maintain the food safety (Li et al., 2015).

Naphthaleneacetic acid is a synthetic form of auxin that improves fruit yield and quality. Studies have shown that NAA affects floral sex ratio and helps with root initiation, apical dominance, fruit setting ratio, fruit falling prevention, vascular tissue differentiation (Mehraj et al., 2015), increased fruit size (Yadav et al., 2017), induced early flowering, augmented flower production and reduced flower and fruit drop (Yamgar and Desai, 1987). In terms of fruit quality, several authors stated that by treating NAA to strawberry fruit, growers can increase anthocyanin accumulation (Yadav et al., 2017), total sugars, ascorbic acid content, and titratable acidity (Kumar and Tripathi, 2009; Bhople et al., 2020). Nonetheless, synthetic auxin can also stimulate leaf senescence and leaf and fruit abscission (Zhu et al., 2011), in addition, it can delay fruit maturation and blooming time (Yadav et al., 2017). The administration time and concentration of NAA, play a pivotal role in crop response (Suman et al., 2017).

One of the most promising new innovation in sustainable contemporary agricultural systems is the use of natural biostimulants considering their natural source and capacity to replace or reduce the use of traditional synthetic hormones. According to the Regulation (EU) 2019/1009, plant biostimulants (PBs) are microbial and non-microbial-products able to stimulate plant nutrition processes independently of the product’s nutrient content with the sole aim of improving one or more of the following characteristics of the plant or the plant rhizosphere: (a) nutrient use efficiency, (b) tolerance to abiotic stress, (c) quality traits, or (d) availability of confined nutrients in the soil or rhizosphere. PBs, whether derived from animal sources (Casadesús et al., 2019), land plant (Lucini et al., 2015; Colla et al., 2017), algae (Golubkina et al., 2022), or microbial sources (Rouphael and Colla, 2018; Cardarelli et al., 2020), are recognized for their positive impact on the hormonal balance of plants. It should not be disregarded that PBs may be a source of phytohormones, or they can modulate plant hormone homeostasis replacing the need of synthetic hormones. For instance, protein hydrolysate (PH) biostimulants are widely used in market nowadays and preferred by farmers due to their environmentally friendly application and effective role in improving crop yield (Colla et al., 2017; Xu and Geelen, 2018). Several studies demonstrated the auxin-like activity of protein hydrolysates on vegetable crops. For instance, Colla et al. (2014) reported an auxin-like activity in coleoptile elongation of corn and shoot, root dry weight, root length, and root area of tomato cuttings from the plant-derived protein hydrolysate “Trainer^®^”. Other authors demonstrated the capacity of the tropical-plant extract biostimulant "Auxym®" in mimicking auxin-like effect on sweet cherry fruits due to the presence of peptides and free amino acids which serve as signaling molecules thus suggesting an alternative model for exogenous synthetic hormones application (Basile et al., 2021). Some authors pointed out that biostimulants derived from vegetal origin, induced a modulation of metabolites related to the regulation of the homeostasis of the auxin pool (Buffagni et al., 2021). In addition to protein hydrolysates, several studies reported that many microorganisms, such as strains belonging to the genera *Pseudomonas*, *Bacillus*, *Pedobacter*, *Pantoea*, *Luteibacter*, *Acinetobacter*, *Lysobacter*, and *Enterobacter*, have plant growth-promoting properties and capability to produce auxin compounds (Khan and Doty, 2009; Agnolucci et al., 2019; Leontidou et al., 2020). However, regulatory constraints can limit the possibility to apply auxin-producing bacterial strains as soil/crop inoculants. Moreover, inconsistent in-field success is a major problem as microbial inoculants often fail to compete with indigenous soil microbes (Shayanthan et al., 2022). Alternatively, auxin-producing bacterial strains can be cultivated in a fermenter for producing auxin-based filtrates as plant biostimulants. Luziatelli et al. (2021) successfully developed a fermentation process with *Enterobacter* sp. strain P-36 for producing a stable auxin-based filtrate. A bacterial culture filtrate containing auxin compounds was successfully tested as foliar spray for increasing fruit weight and marketable yield of greenhouse tomato (Rouphael et al., 2021).

Omics studies, encompassing transcriptomics and metabolomics among other powerful techniques, play a pivotal role in unravelling the molecular mechanisms underlying biostimulation, providing invaluable information into the regulatory networks and metabolic pathways involved into the plant response, respectively. For example, transcriptomics has been employed to decipher global gene expression patterns, uncovering the molecular mechanisms modulated by biostimulants in enhancing plant growth, development, and/or stress tolerance (González-Morales et al., 2021; Ali et al., 2022; Baghdadi et al., 2022). Metabolomics has been employed to comprehensively analyze and identify variations relative to small metabolites, offering insights into the metabolic processes, pathways, and biochemical changes occurring in response to plant biostimulants (Lucini et al., 2015; Bonini et al., 2020a). In particular, a metabolomics investigation indicated that a foliar application of five root-promoting PHs to tomato cuttings stimulated greatly the accumulation of the IAA precursors 4-(indol-3-yl) butanoate (IBA) and tryptamine.

To be competitive, the greenhouse production of strawberries requires higher quanti-qualitative standards to satisfy the needs of the consumer and large-scale distribution and reducing production costs. This need has created a continuous search by the farmers for products that can help them in pursuing these objectives and offer the consumers healthier products respecting food safety and the environment. Starting from the above considerations, we hypothesized that biostimulants with auxin-like activity can replace the synthetic auxins improving the quali-quantitative traits of strawberry by modulating plant metabolism. Consequently, the primary objective of this study was to assess the efficacy of two bio-based products, namely an auxin-enriched bacterial filtrate and a vegetal-derived protein hydrolysate. These alternatives were investigated as substitutes for commonly used synthetic auxins (NAA and naphthaleneacetamide; NAD), with the aim of enhancing both the production and fruit quality of greenhouse-grown strawberry plants. In particular, our primary aims encompassed a comprehensive evaluation of these biostimulants’ influence on the growth, development, and overall performance of the plants. Our investigation also extended into the ionomics and metabolomics responses triggered by the application of these biostimulants. To the best of our knowledge, this study represents the first exploration of biostimulants with auxin-like activity and their impact on strawberry crops, providing the foundational evidence necessary for informed decisions in the pursuit of sustainable and productive greenhouse strawberry cultivation.

## 2 Materials and methods

### 2.1 Plant material, experimental design, and growing conditions

The trial was carried out in a polyethylene greenhouse at the experimental farm ‘Nello Lupori’, University of Tuscia, Viterbo, Italy. The average day/night air temperatures were 24± 0.8/16± 0.9°C. Plants were grown in polyethylene bags, white on the outside and black on the inside, containing 33 litres each (22 × 100 cm). The substrate had the following characteristics: 50% perlite (granulometry 1-2 mm) and 50% coconut fiber (v:v ratio), pH of 6.5, and EC of 0.7 mS/cm. The substrate was saturated with a nutrient solution before planting. The nutrient solution contained the following nutrients: 8.5 mM N-NO_3_, 2.0 mM S, 1.0 mM P, 3.2 mM K, 4.0 mM Ca, 0.9 mM Mg, 20 μM Fe, 9 μM Mn, 0.3 μM Cu, 1.6 μM Zn, 20 μM B, and 0.3 μM Mo. The pH of the nutrient solution was 5.5 ± 0.2 and the EC was 1.4 ± 0.1 mS/cm. Deionized water was used for the preparation of nutrient solution.The transplant of rooted strawberry plug plants (*Fragaria* × *ananassa* Duch.– cv ‘Nabila’; Salvi Vivai, Ferrara, Italy) was carried out on 02/10/2019 at a plant density of 8.3 plants/m^2^ (10 plants per bag arranged as double row; bag rows were spaced 1.2 m apart). The cultivar ‘Nabila’ is widely used in Mediterranean countries due to the very early production and good quali-quantitative traits of fruits (high fruit size, bright red color, excellent texture, and high productivity). Nutrient solution was pumped from independent supply tanks through a drip irrigation system, with one emitter per plant of 2 L h^-1^ flow rate. The duration of each irrigation event was tuned to provide at least 35% of the nutrient solution draining from the pots.

Five foliar treatments were tested as follow: control; bacterial filtrate; synthetic auxins; and vegetal-derived protein hydrolysate. The vegetal-derived protein hydrolysate Trainer^®^ (Hello Nature S.p.a., Rivoli Veronese, Italy) was applied at a dose of 5 ml L^-1^. Trainer^®^ was made by enzymatic hydrolysis of proteins from legume seeds. According to Colla et al. (2015) and Lucini et al. (2015), Trainer^®^ contained primarily soluble peptides and free amino acids (310 g kg^-1^). The synthetic auxin Auxyger^®^ LG (L. Gobbi s.r.l., Campo Ligure, Italy) was applied at a dose of 0.5 ml L^-1^; it is a liquid plant growth regulator based on pure NAD at 16.9 g L^-1^ and pure NAA at 6.7 g L^-1^. The bacterial filtrate Capxium^®^ (Atens, Tarragona, Spain) was a commercial product obtained by fermentation with a proprietary strain of the *Pantoea* genera in a substrate rich in tryptophan to maximize the production of indole-3-acetic acid. Capxium^®^ was applied at a rate of 5 ml L^-1^. Control treatment was foliarly sprayed with pure water. Foliar treatments started at flowering stage on 29/01/2020 (119 days after transplanting -DAT) and were repeated three more times: 7/02/2020 (128 DAT), 17/02/2020 (138 DAT), and 27/02/2020 (148 DAT). Tested products were uniformly sprayed with a 16-L stainless steel sprayer called Vibi Sprayer (Volpi, Piadena, Italy). Treatments were arranged in a randomized complete block design with 5 replicates. Each plot was composed by one bag with 10 plants. All early-forming runners were removed from strawberry plants in order to prolong the fruit harvest. Fungal diseases were controlled with 2 foliar sprays of a fungicide containing cyprodinil and fludioxonil (Switch^®^; Syngenta, Milano, Italy) at a rate of 0.8 mg L^-1^ while pests were controlled with 2 foliar sprays of an insecticide containing abamectin (Vertimec EC; Syngenta, Milano, Italy) at a rate of 0.4 ml L^-1^.

### 2.2 Plant production and fruit quality analysis

Fruit harvest began on 03/03/2020 (153 DAT), followed by 06/03/2020 (156 DAT), 09/03/2020 (159 DAT), 12/03/2020 (162 DAT), 20/03/2020 (170 DAT), 26/03/2020 (176 DAT), 03/04/2020 (184 DAT), 10/04/2020 (191 DAT), 16/04/2020 (197 DAT), and 22/04/2020 (209 DAT). In each harvest, fruits were collected separately in each plot and sorted in marketable (red fruits), and unmarketable (fruits having a diameter lower than 25 mm, fruits rotten and/or deformed). Fruits of two groups were counted and weighted separately. Marketable fresh mean weight was determined dividing the marketable fruit weight by the number of marketable fruits. Early yield was calculated considering the marketable fruits collected from the first harvest (153 DAT) to the sixth harvest (176 DAT). Eight marketable fruits per experimental unit were selected on the middle of harvesting period (176 DAT) for fruit quality analyses. Fruits were oven-dried at 65 °C until constant weight for determining fruit dry matter. Fruit firmness (kg cm^-2^) was determined using a penetrometer (Bertuzzi FT 011; Milan, Italy), fitted with a 6 mm-diameter round-head probe. Then the fresh strawberry fruits were homogenized in a blender (2 L capacity; Waring HGB140, CA, USA) for one minute at low speed. The slurry was filtered through a two-layer cheesecloth, where the total soluble solids (TSS; expressed in °Brix) content was read with an electronic Atago N1 refractometer (Atago Co. Ltd., Tokyo, Japan).

At the final harvest (209 DAT), the Soil Plant Analysis Development (SPAD) index was measured on 20 topmost fully expanded leaves per plot with the SPAD-502 instrument (Konica Minolta Europe). After SPAD readings, shoots were harvested and oven-dried at 65 °C until constant weight for determining shoot dry weight.

Fruit mineral assessment was performed on dry samples of marketable fruits harvested at 176 DAT. Dried fruits were ground separately in a Wiley mill to pass through a 20-mesh screen, then 0.5 g of the dried plant tissues were analyzed for the following mineral elements: N, P, K, Ca, Mg, Fe, Mn, Zn, Cu, B, Mo, Ni, Na, Al, As, Ba, Be, Cd, Co, Cr, Pb, Sb, Se, Sn, Ti, Tl, and V. Nitrogen concentration in the plant tissues was determined after mineralization with sulfuric acid by ‘Kjeldahl method’ (Bremner, 1965) while the other elements were determined by dry ashing at 400 °C for 24 h, dissolving the ash in 1:20 HNO_3_, and assaying the solution obtained using an inductively coupled plasma emission spectrophotometer (ICP Iris; Thermo Optek, Milano, Italy) (Horneck and Miller, 1998).

### 2.3 Fruit metabolomics

On 26/03/2020 (176 DAT), 5 marketable fruits per plot were harvested and immediately frozen with liquid nitrogen and stored at -20°C. Fruits were grinded in liquid nitrogen for metabolomic analysis at oloBion Laboratory (Barcelona, Spain). Metabolites were extracted in acidified 80% methanol, as previously reported (Bonini et al., 2020a). The samples were extracted by Ultra- Turrax (Ika T-25; Staufen, Germany), centrifuged and filtered through a 0.22 µm cellulose membrane into vials for analysis. A UHPLC chromatographic system coupled to a quadrupole-time-of-flight mass spectrometer (UHPLC/QTOF-MS) was used for the untargeted screening of metabolites (Bonini et al., 2020b). The polar metabolites were separated at 45°C on a Water Acquity UPLC BEH C18 column (100 mm length x 2.1 mm id; 1.7 µm particle size) equipped with an additional Water Acquity VanGuard BEH C18 pre-column (5 mm x 2.1 mm id; 1.7 µm particle size) using (A) water with 0.1% formic acid and (B) acetonitrile with 0.1% formic acid as A and B mobile phases respectively, with a gradient elution starting at 0 min with 0.5% B, 0-0.1 min 0.5% B, 0.1-10 min 80% B, 10-10.1 min 99.5% B, 10.1-12 min 99.5% B, 12-12.1 min 0.5% B, 12.1-14.4 min 0.5% B, and 14.4-14.5 min 0.5% B. Mobile phase flow rate was set at 0.3 ml/min, and the injection volume was 15µl. The sample temperature was maintained at 4 °C. Following the separation, the flow was introduced by positive mode electrospray ionization (ESI) into the mass spectrometer with the following parameters: capillary voltage, ± 3 kV; gas temperature, 250°C; drying gas (nitrogen), 13 L/min, nebulizer gas (nitrogen), 50 psi; sheath gas temperature, 315°C; sheath gas flow (nitrogen), 12 L/min and acquisition rate, 1 spectra/s.

For metabolite identification, MS/MS spectra were collected at collision energies of 10, 20, and 50 eV with an acquisition rate MS^1^ of 4 spectra/s (100 ms) and an acquisition rate for MS/MS of 3 spectra/s (77 ms) with 4 precursor ions per cycle.

### 2.4 Statistical analysis

Agronomic and mineral data were subjected to analysis of variance (ANOVA) and Duncan’s test (p = 0.05) to determine significant differences between treatments. Before analysis of variance, homogeneity of variance was assessed using Levene’s Test for equality of variances, and the percentage data of early marketable yield was subjected to arcsine transformation to make the distribution normal. All statistical analysis were performed using the SPSS software package, (SPSS 10 for Windows 2001). Metabolomic data was processed by oloMAP 2.0^2^ created by oloBion Company (Bonini et al., 2020b).

## 3 Results

### 3.1 Growth, yield and fruit quality

**Figure 1:**
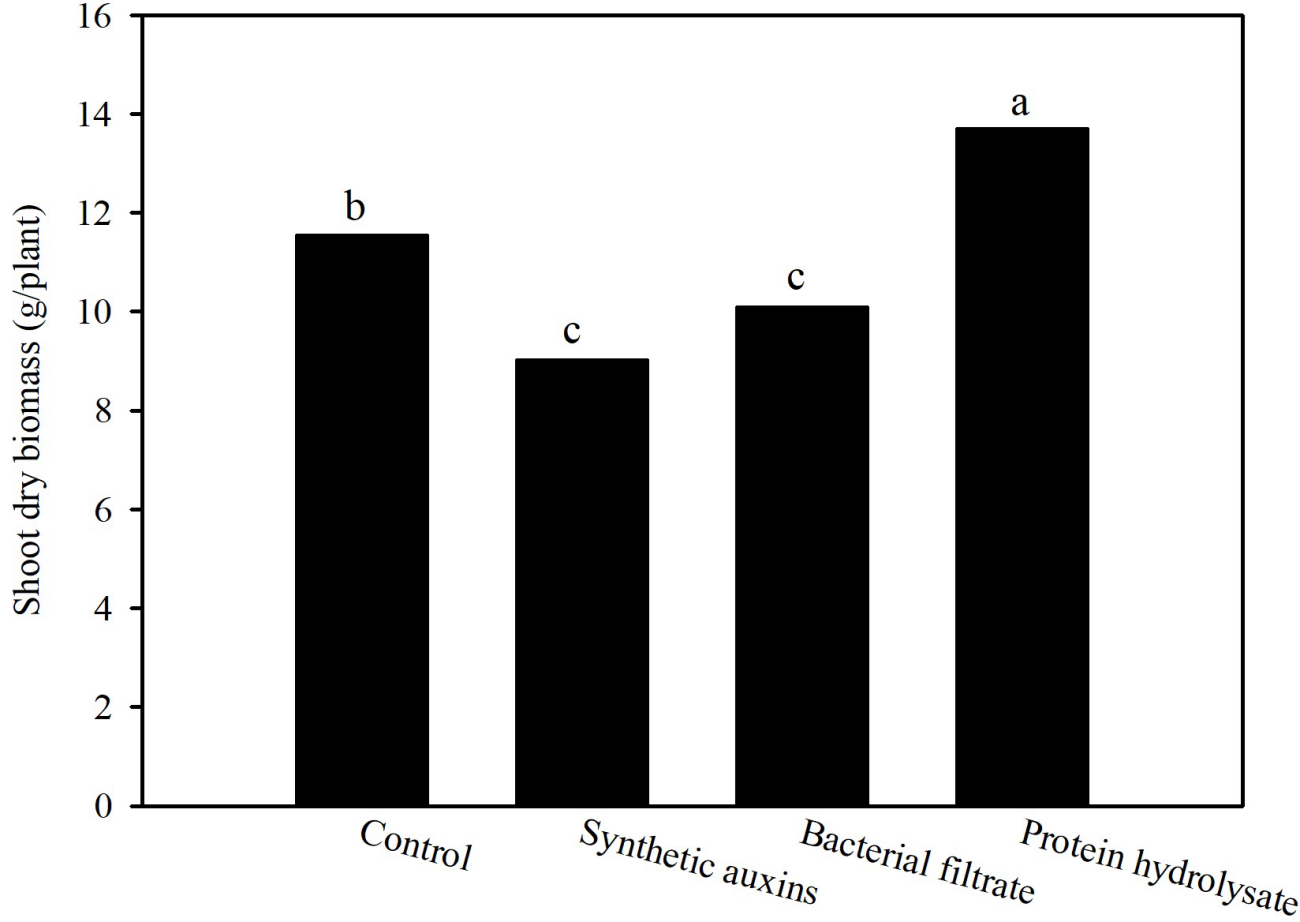
Effect of treatments on shoot dry biomass of strawberry plants at the end of the trial. Different letters correspond to statistically different values for p=0.05 (Duncan’s test).

The shoot dry biomass at the end of the cycle was the highest in plants treated with protein hydrolysate (PH), followed by the control plants, and plants treated with Auxyger^®^ LG (SA), and bacterial filtrate (BF) (**Figure 1**). No statistically significant difference was found regarding SPAD index of leaves at the end of the trial (avg. 54.6; data not shown).

**Figure 2:**
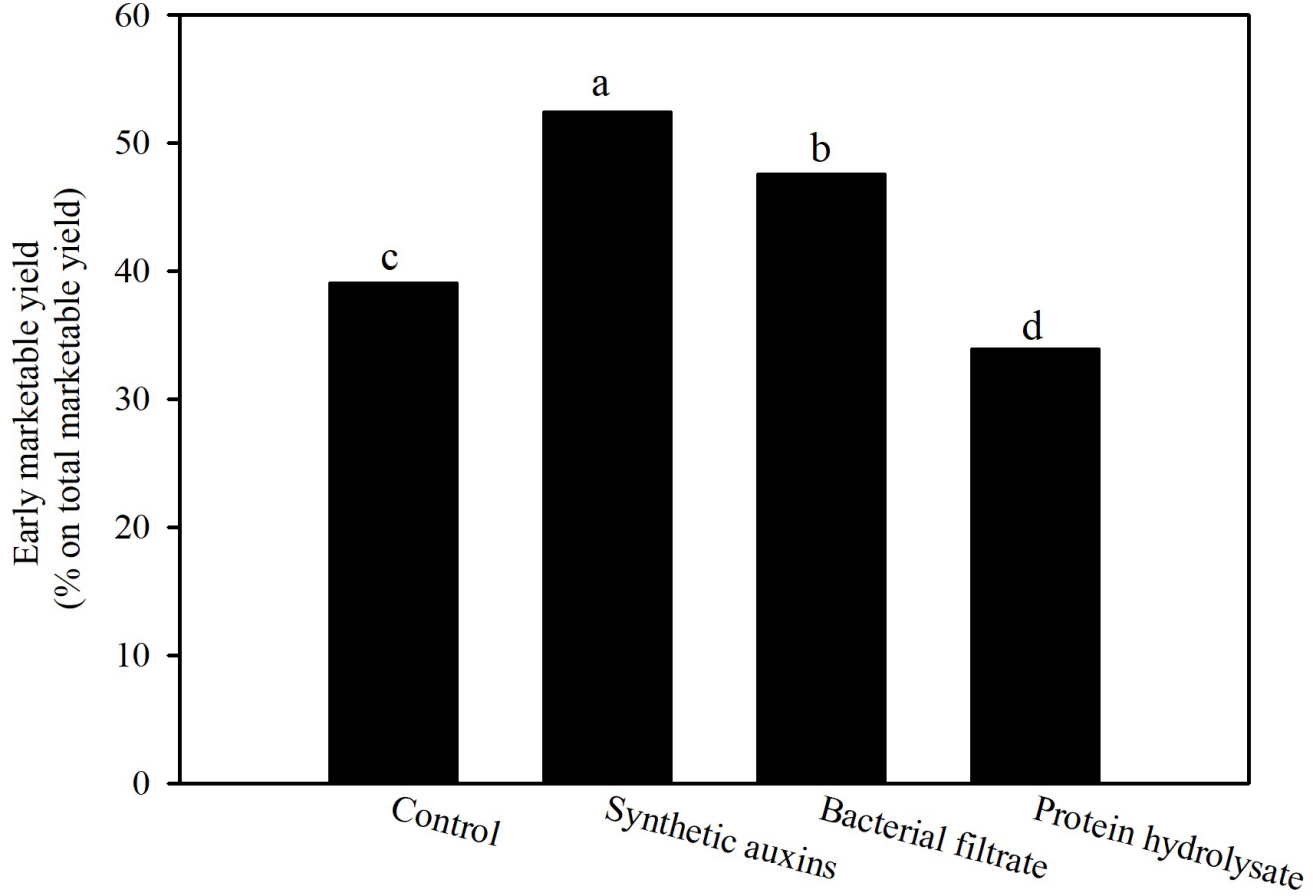
Effect of treatments on percentage of early marketable yield on total marketable yield of strawberry plants. Data are back transformed from arcosin transformation. Different letters correspond to statistically different values for p=0.05 (Duncan’s test).

Strawberry early production (first 6 harvests) was significantly influenced by treatments with the highest values recorded in plants treated with BF, and with SA, followed by the control treatment and PH (**Table 1**). Total marketable yield was highest in plants treated with PH followed by the untreated plants, and plants treated with BF with no significant difference among them, whereas the lowest value was obtained in plants treated with SA (**Table 1**). The percentage of early marketable production out of the total marketable yield was the highest with the foliar treatments of SA (52.4%), followed with plants treated with BF (47.6%), while control plants (39.9%) and especially PH treated plants (33.9%) provided the lowest values (**Figure 2**). Because no significant differences were recorded for unmarketable yield, total marketable yield followed the same behavior of total yield with the highest value in PH treatment (**Table 1**). The differences on total marketable yield were attributed to changes of fruit numbers and not to fruit mean weight (**Table 1**).

**Table 1:** Effect of treatments on early marketable (Early M), total marketable (Total M), unmarketable (U), and total yield (Total), and number and mean weight of marketable fruits in strawberry plants.

Treatment	Fruit yield (g/plant)				Marketable fruits	
	Early M	Total M	U	Total	Number (n./plant)	Mean weight (g/fruit)
Control	103.6 b	265.1 b	51.1	316.1 b	14.9 b	17.8
Synthetic auxins	119.7 a	226.6 c	44.9	271.5 c	12.7 c	17.9
Bacterial filtrate	120.1 a	255.5 b	49.2	304.7 b	14.2 b	18.0
Protein hydrolysate	102.1 b	300.3 a	48.1	348.4 a	16.6 a	18.1
Significance	**	***	ns	**	***	ns

ns, **, *** Nonsignificant or significant at p ≤ 0.01 and 0.001, respectively. Different letters in the same column correspond to statistically different values for p=0.05 (Duncan’s test).

Fruit firmness was significantly higher in plants treated with Trainer^®^ (PH) compared to SA treatment (**Table 2**); BF treatment and control exhibited intermediated values which were no significant different from the other treatments (control, SA). Fruit dry matter was not significantly affected by treatments (**Table 2**). The content of soluble solids in fruits treated with SA was lower compared to control and PH treatment while BF treated fruits had intermediate values (**Table 2**).

**Table 2:** Effect of treatments on quality traits of strawberry fruits.

Treatment	Fruit firmness (kgf/cm^2^)	Dry matter (%)	Soluble solids (°Brix)
Control	1.85 ab	6.24	5.15 a
Synthetic auxins	1.61 b	6.70	4.54 b
Bacterial filtrate	1.78 ab	6.29	5.12 ab
Protein hydrolysate	1.87 a	6.76	5.22 a
Significance	*	ns	*

ns, * Nonsignificant or significant at p ≤ 0.05, respectively. Different letters in the same column correspond to statistically different values for p=0.05 (Duncan’s test).

### 3.2 Mineral profiling

Treatments affected only the nitrogen (N) concentration in strawberry leaves, while no significant differences were found for the other macronutrients (**Table 3**). The highest N content was recorded in leaves treated with PH compared to plants treated by BF, SA, and untreated plants (**Table 3**). Trace elements were also affected by the different treatments (**Table 3**). Zinc (Zn) had a significantly higher value in plant leaves treated with BF in comparison with leaves treated by PH, with no significant differences with leaves treated by SA and control plants (**Table 3**). Copper (Cu) had the highest value in leaves treated with SA compared to all other treatments and untreated leaves (**Table 3**). Boron (B) element was significantly higher in leaves treated with SA in comparison to leaves treated by BF and PH with no significant difference compared to control leaves (**Table 3**). Control leaves had a significantly higher B concentration than BF and PH treated plant leaves (**Table 3**). Nickel (Ni) concentration was highest in leaves of the untreated plants compared to all other treatments (**Table 3**). Selenium (Se) concentration was significantly highest in leaves treated with PH, followed by synthetic auxins and untreated control with no significant differences with BF treated leaves (**Table 3**). BF treated leaves had a significantly higher (Se) concentration compared to untreated leaves (**Table 3**). The heavy metal cadmium (Cd) element was significantly higher in leaves of control plants and the ones treated with BF in comparison to plant leaves treated with SA, with no significant difference to leaves treated with PH (**Table 3**). Antimony (Sb), Thallium (TI), Vanadium (V), Cobalt (Co), Arsenic (As), Beryllium (Be) trace elements had significantly the highest values in untreated plant leaves, in comparison to all treated plant leaves (**Table 3**).

**Table 3:** Effect of treatments on the mineral composition of strawberry leaves.

Element	Control	Synthetic auxins	Bacterial filtrate	Protein hydrolysate	Significance
	———————— g/kg d.wt.—————————				
N	22.341 b	21.784 b	22.419 b	25.384 a	**
P	3.843	4.120	4.022	4.117	ns
K	24.096	25.293	25.229	23.842	ns
Ca	14.584	14.063	13.361	14.148	ns
Mg	3.175	3.285	3.228	3.121	ns
	———————— mg/kg d.wt.—————————				
Fe	78.156	77.194	81.486	82.702	ns
Mn	265.653	302.317	274.683	270.110	ns
Zn	47.762 ab	51.060 ab	45.704 a	42.963 b	*
Cu	223.630 b	283.558 a	201.442 b	198.453 b	**
B	266.775 a	278.046 a	192.022 b	191.758 b	**
Mo	0.686	0.641	0.865	0.801	ns
Al	55.989	88.804	97.516	72.831	ns
Ba	63.065	65.611	70.340	59.582	ns
Ni	6.792 a	1.850 b	1.181 b	1.770 b	***
Se	0.529 c	0.773 b	0.793 ab	0.883 a	**
Cd	0.243 a	0.185 b	0.240 a	0.194 ab	*
Cr	0.250	0.098	0.567	0.141	ns
Pb	0.172	0.237	0.374	0.167	ns
Sn	0.810	0.924	0.990	0.939	ns
Ti	0.367	0.550	0.709	0.410	ns
Sb	0.016 a	0.014 b	0.013 b	0.014 b	***
TI	9.769 a	8.566 b	8.273 b	8.510 b	***
V	10.010 a	8.777 b	8.477 b	8.720 b	***
Co	0.965 a	0.846 b	0.817 b	0.840 b	***
As	3.679 a	3.225 b	3.115 b	3.204 b	***
Be	0.181 a	0.159 b	0.153 b	0.158 b	***

ns, *, **, *** Nonsignificant or significant at p ≤ 0.05, 0.01 and 0.001, respectively. Different letters in the same row correspond to statistically different values for p=0.05 (Duncan’s test).

Macronutrients concentration (N, P, K, Ca, Mg) in strawberry fruits was not affected by treatments whereas significant differences were recorded for some trace elements (**Table 4**). Boron concentration was significantly highest in control treatment compared to SA and PH treatments with no significant difference with bacterial filtrate treatment. No significant difference was found between B concentration in fruits of plants treated with SA and PH. Aluminium concentration was highest in fruits of plants treated with BF, compared to fruits of plants treated with SA with no significant difference in comparison with fruits of plants treated with PH and control. Nickel concentration was significantly higher in fruits of control plants and SA treated plants in comparison with PH and BF treated plants with no significant differences among the two latter. Cadmium concentration was significantly higher in fruits of untreated plants in comparison to fruits treated by SA and PH while BF showed intermediate values with no significant differences from the other treatments. Lead (Pb) concentration was significantly highest in untreated plants, followed by fruits treated with BF and then by PH and SA, with no significant differences among these two latter. Titanium (Ti) concentration in fruits treated by BF and PH had a significantly higher value than fruits treated by SA with no significant difference with fruits of untreated plants. Fruits treated by SA had no significant difference with fruits of untreated plants. No significant differences were recorded for Sb, TI, V, Co, As and Be.

**Table 4:** Effect of treatments on the mineral composition of strawberry fruits.

Element	Control	Synthetic auxins	Bacterial filtrate	Protein hydrolysate	Significance
	———————— g/kg d.wt.—————————				
N	18.316	17.193	18.524	19.582	ns
P	3.488	3.687	3.443	3.285	ns
K	19.614	18.616	20.472	19.276	ns
Ca	8.239	8.788	7.571	7.242	ns
Mg	1.769	1.676	1.667	1.554	ns
	———————— mg/kg d.wt.—————————				
Fe	49.244	37.002	44.672	39.218	ns
Mn	75.329	59.431	72.974	56.067	ns
Zn	22.397	20.579	21.334	20.890	ns
Cu	9.512	6.511	8.711	8.095	ns
B	45.981 a	30.671 b	40.942 a	33.814 b	**
Mo	0.323	0.304	0.303	0.267	ns
Al	336.406 ab	285.838 b	440.211 a	375.540 ab	**
Ba	9.282	9.184	9.137	9.404	ns
Ni	5.071 a	1.761 b	3.605 a	1.514 b	***
Se	1.069	0.908	1.045	0.916	ns
Cd	0.061 a	0.043 b	0.052 ab	0.043 b	*
Cr	0.523 a	0.315 ab	0.097 bc	0.061 c	***
Pb	0.150 a	0.027 c	0.076 bc	0.034 c	**
Sn	1.113	0.959	1.012	0.949	ns
Ti	0.242 ab	0.139 b	0.278 a	0.329 a	*
Sb	0.015	0.015	0.014	0.015	ns
TI	9.242	9.491	8.833	9.346	ns
V	9.470	9.725	9.051	9.577	ns
Co	0.913	0.937	0.872	0.923	ns
As	3.480	3.574	3.326	3.519	ns
Be	0.171	0.176	0.164	0.173	ns

ns, *, **, *** Nonsignificant or significant at p ≤ 0.05, 0.01 and 0.001, respectively. Different letters in the same row correspond to statistically different values for p=0.05 (Duncan’s test).

### 3.3 Metabolomic analysis

**Figure 3:**
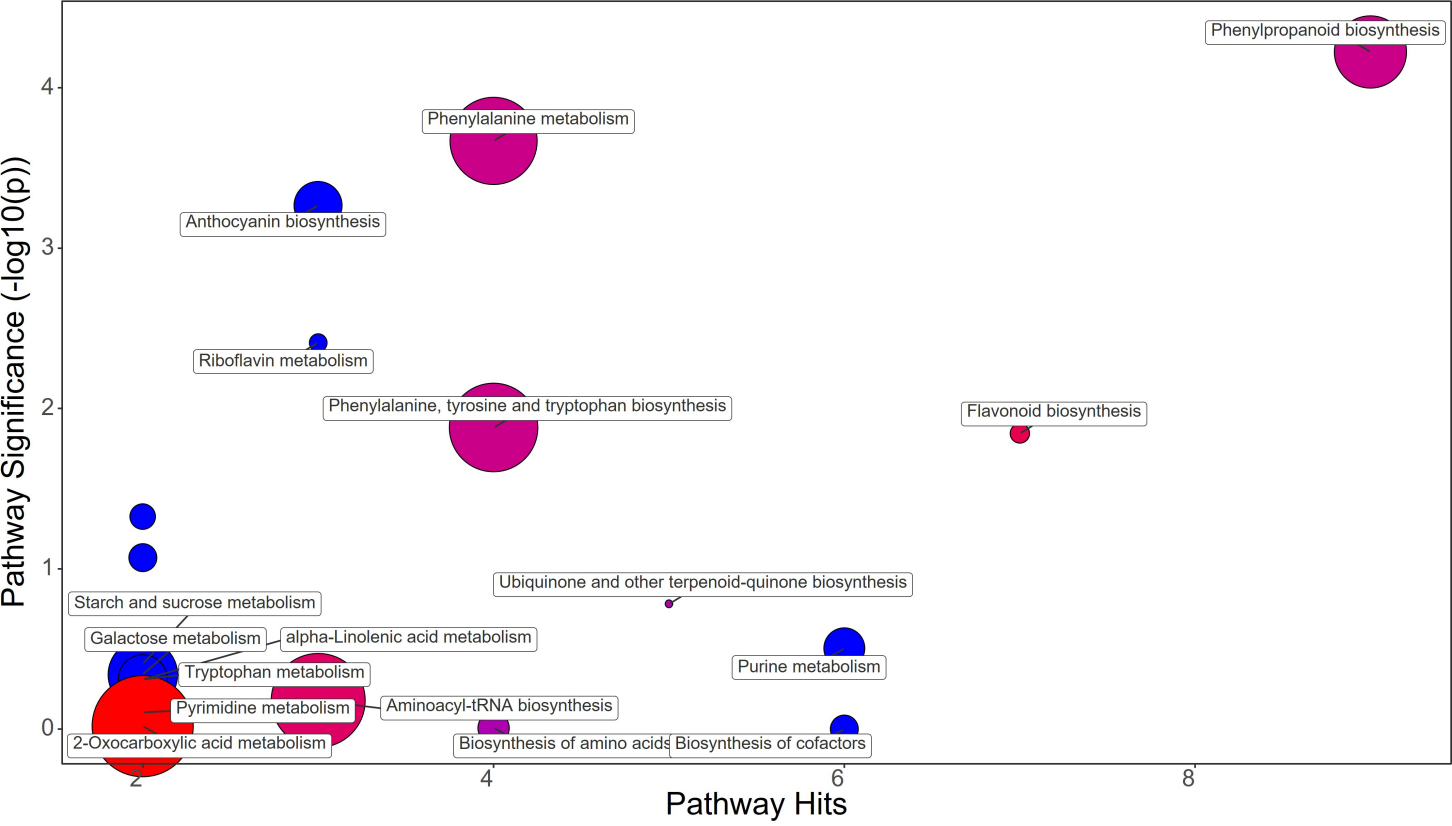
Chemical similarity enrichment analysis (ChemRICH) of statistically different annotated metabolites in protein hydrolysate treated leaves compared to untreated control of strawberry plants. Pathway significance (-log10(p)) is plotted on the y-axis, while the pathway hits are represented on the x-axis. Clusters are color-coded to indicate the proportion of compounds that have increased (in red), decreased (in blue), or exhibited mixed changes (in purple).

The different treatments applied to the plants clearly showed distinctive metabolomic profiles at leaf level. Fold change (FC) values were calculated comparing the treatment group against the control group. A value greater than 1 means an increase while a value below 1 means a decrease of the metabolite. Protein hydrolysate (PH) treatment induced the great increase in Phenylalanine (FC=21.77) and Tyrosine (FC=2.37), contrasting with the decrease in Chorismate (FC=0.53) and Tryptophan (FC=0.84) abundance in strawberry leaves (**Figure 3**; **Supplementary File 1**). The phenylpropanoid pathway was downregulated showing a decrease in Ferulate (FC=0.76) and Syringin (FC=0.55). On the other hand, the flavonoid biosynthesis pathway showed an increase mostly due to Catechin (FC=1.37) and Kaempferol (FC=1.25), despite the decrease in Gallocatechin (FC=0.83). Purine metabolism also featured the decrease in Guanine (FC=0.56), dADP (FC=0.59), Adenosine (FC=0.53), and Deoxyadenosine (FC=0.20). The drop of Anthocyanin was also revealed by the decrease in Delphinidin 3-O-glucoside (FC=0.75) and Pelargonidin 3-O-glucoside (FC=0.07) (**Supplementary File 1**).

**Figure 4:**
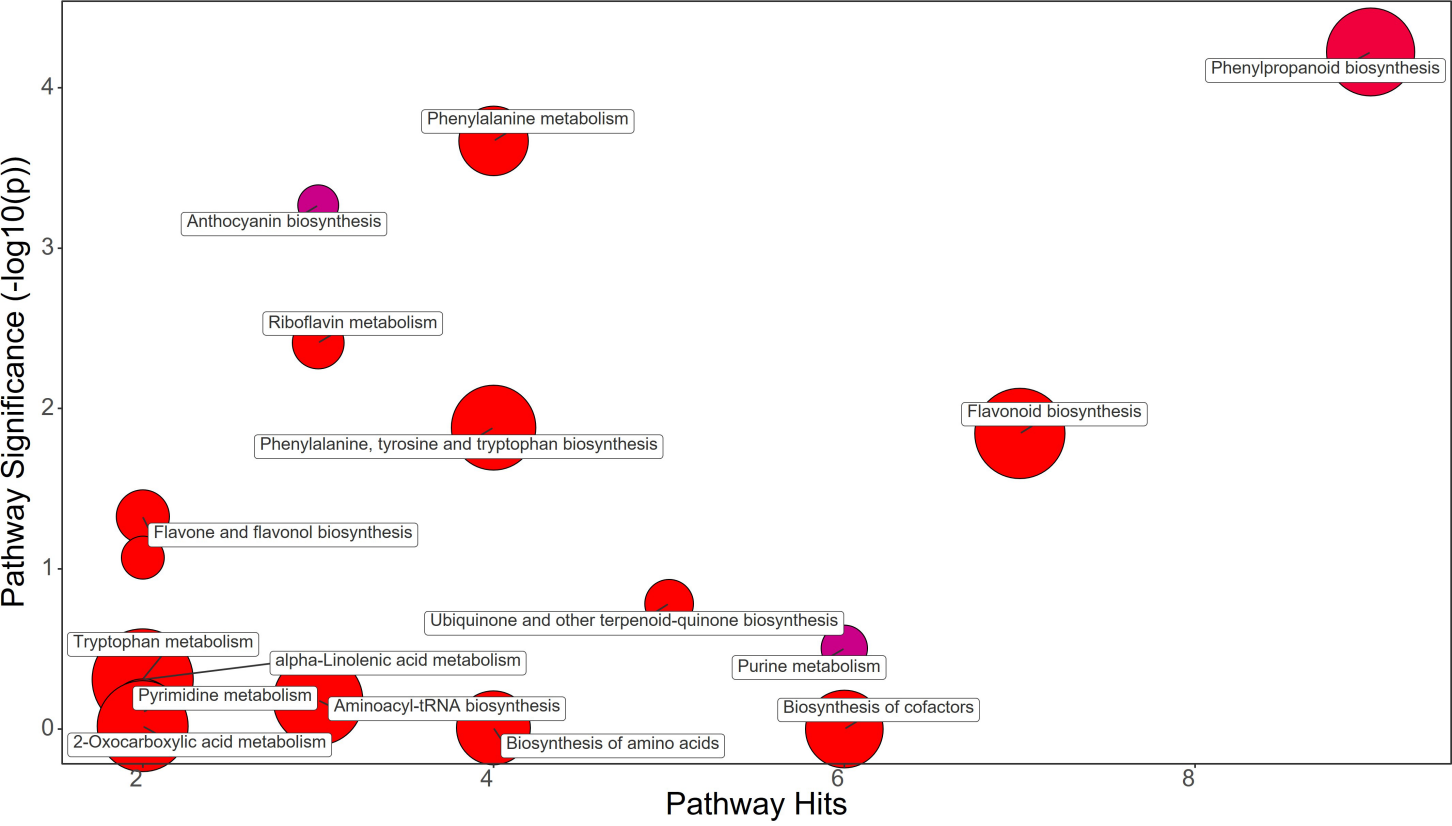
Chemical similarity enrichment analysis (ChemRICH) of statistically different annotated metabolites in synthetic auxins treated leaves compared to untreated control of strawberry plants. Pathway significance (-log10(p)) is plotted on the y-axis, while the pathway hits are represented on the x-axis. Clusters are color-coded to indicate the proportion of compounds that have increased (in red), decreased (in blue), or exhibited mixed changes (in purple).

**Figure 5:**
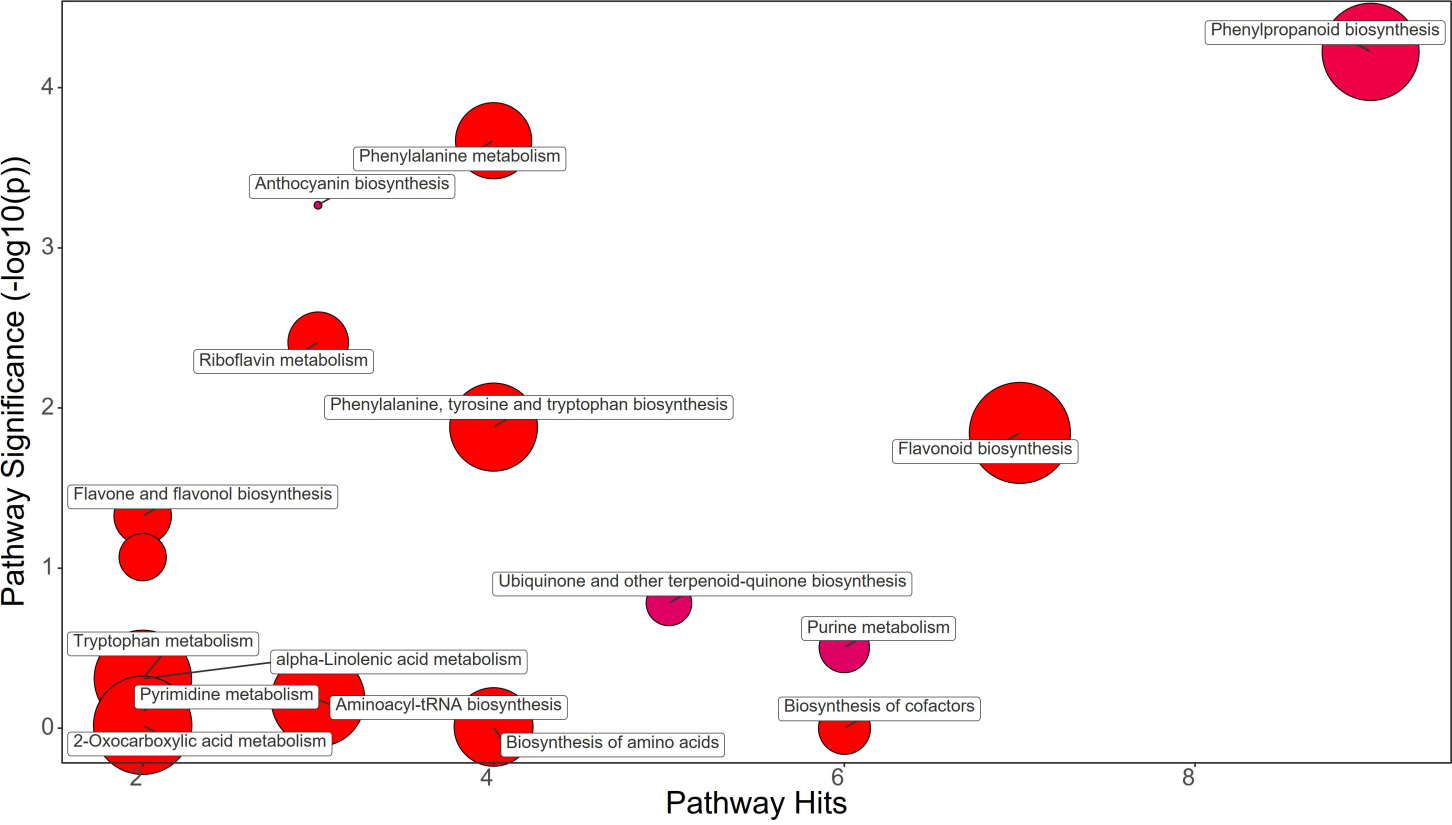
Chemical similarity enrichment analysis (ChemRICH) of statistically different annotated metabolites in bacterial filtrate treated leaves compared to untreated control of strawberry plants. Pathway significance (-log10(p)) is plotted on the y-axis, while the pathway hits are represented on the x-axis. Clusters are color-coded to indicate the proportion of compounds that have increased (in red), decreased (in blue), or exhibited mixed changes (in purple).

Synthetic auxins treatment showed an increase in Phenylalanine (FC=5.09), together with Phenylacetaldehyde (FC=1.32), Tyrosine (FC=2.33), Chorismate (FC=1.69), and Tryptophan (FC=3.17) in strawberry leaves (**Figure 4**; **Supplementary File 1**). The phenylpropanoid pathway featured the increase in Ferulate (FC=11.44), Scopoletin (FC=6.39), Coniferyl alcohol (FC=1.83), and Coumaroyl quinic acid (FC=1.72), and a decrease in Syringin (FC=0.69). Contrasting results were obtained for Purine metabolism with the up-regulation of Xanthosine (FC=1.63) and the downregulation of dADP (FC=0.84) as well as for Anthocyanin metabolism showing the increase in Delphinidin (FC=1.17) and a decrease in Delphinidin 3-O-glucoside (FC=0.75) and Pelargonidin 3-O-glucoside (FC=0.38). More consistent was the enhancement of the flavonoid biosynthesis pathway with increases in Naringenin (FC=9.87), Kaempferol (FC=2.65), Gallocatechin (FC=1.68), and Delphinidin (FC=1.17) (**Supplementary File 1**). Bacterial filtrate treatment caused an increase in Phenylalanine (FC=4.52), as well as Phenylacetaldehyde (FC=1.83), Tyrosine (FC=2.52), Chorismate (FC=1.48), and Tryptophan (FC=2.30) in strawberry leaves (**Figure 5**; **Supplementary File 1**). The phenylpropanoid pathway showed the up regulation of Ferulate (FC=11.68), Scopoletin (FC=6.98), Coniferyl alcohol (FC=1.49), and Coumaroyl quinic acid (FC=2.19), with the depletion in Syringin (FC=0.83). Regarding Purine metabolism it also featured the increase in Xanthosine (FC=2.14) and the decrease in dADP (FC=0.89). Concerning the flavonoid biosynthesis pathway compounds like Catechin (FC=11.15), Naringenin (FC=7.30), Kaempferol (FC=1.88), Gallocatechin (FC=1.46), and Delphinidin (FC=1.44) resulted in a net increase. In this case also Anthocyanin metabolism showed less consistency about the sense of change, with more abundance in Delphinidin (FC=1.44), Delphinidin 3-O-glucoside (FC=1.64), but depletion in Pelargonidin 3-O-glucoside (FC=0.07) (**Supplementary File 1**).

**Figure 6:**
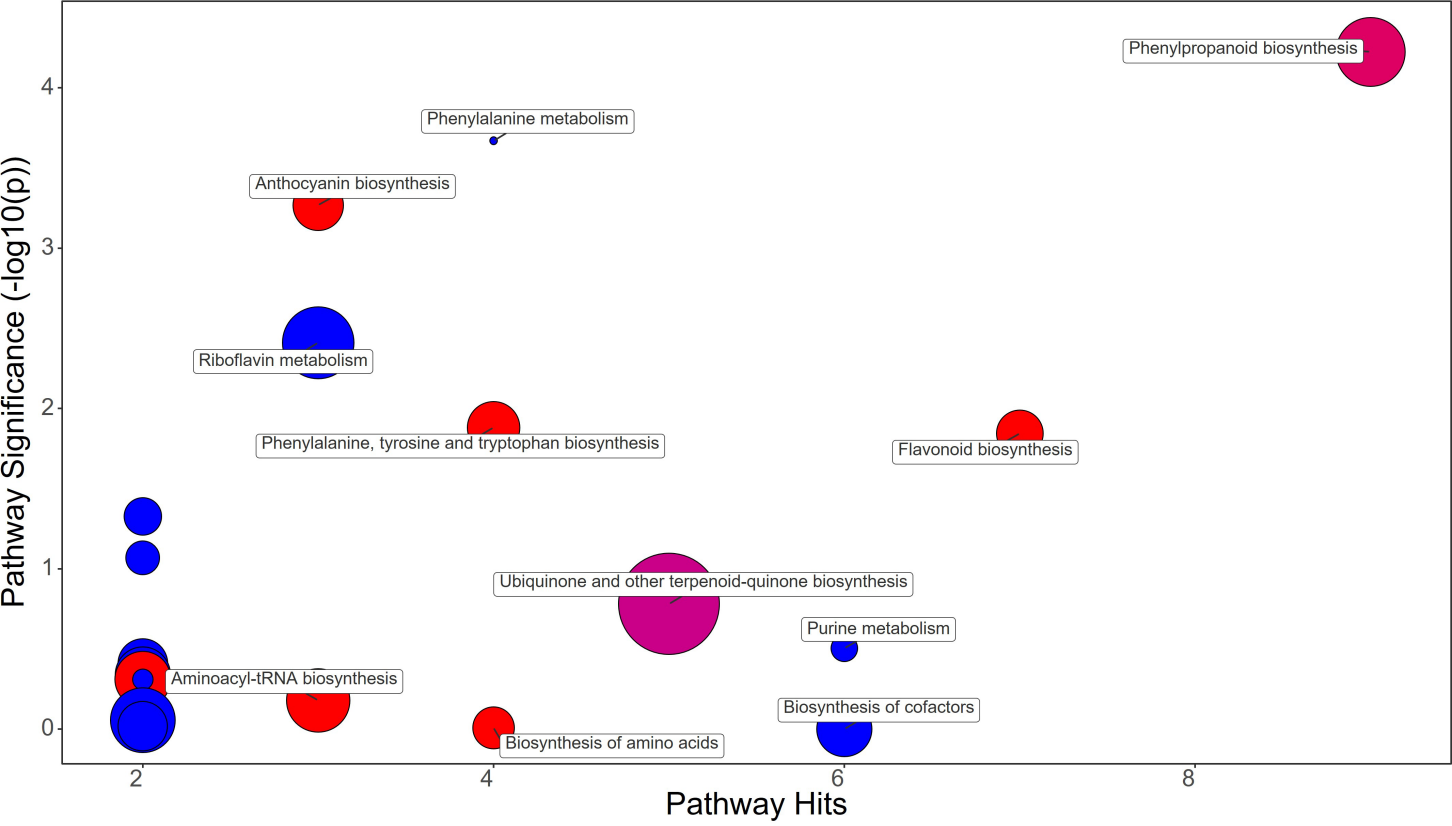
Chemical similarity enrichment analysis (ChemRICH) of statistically different annotated metabolites in bacterial filtrate treated fruits compared to untreated control of strawberry plants. Pathway significance (-log10(p)) is plotted on the y-axis, while the pathway hits are represented on the x-axis. Clusters are color-coded to indicate the proportion of compounds that have increased (in red), decreased (in blue), or exhibited mixed changes (in pink).

**Figure 7:**
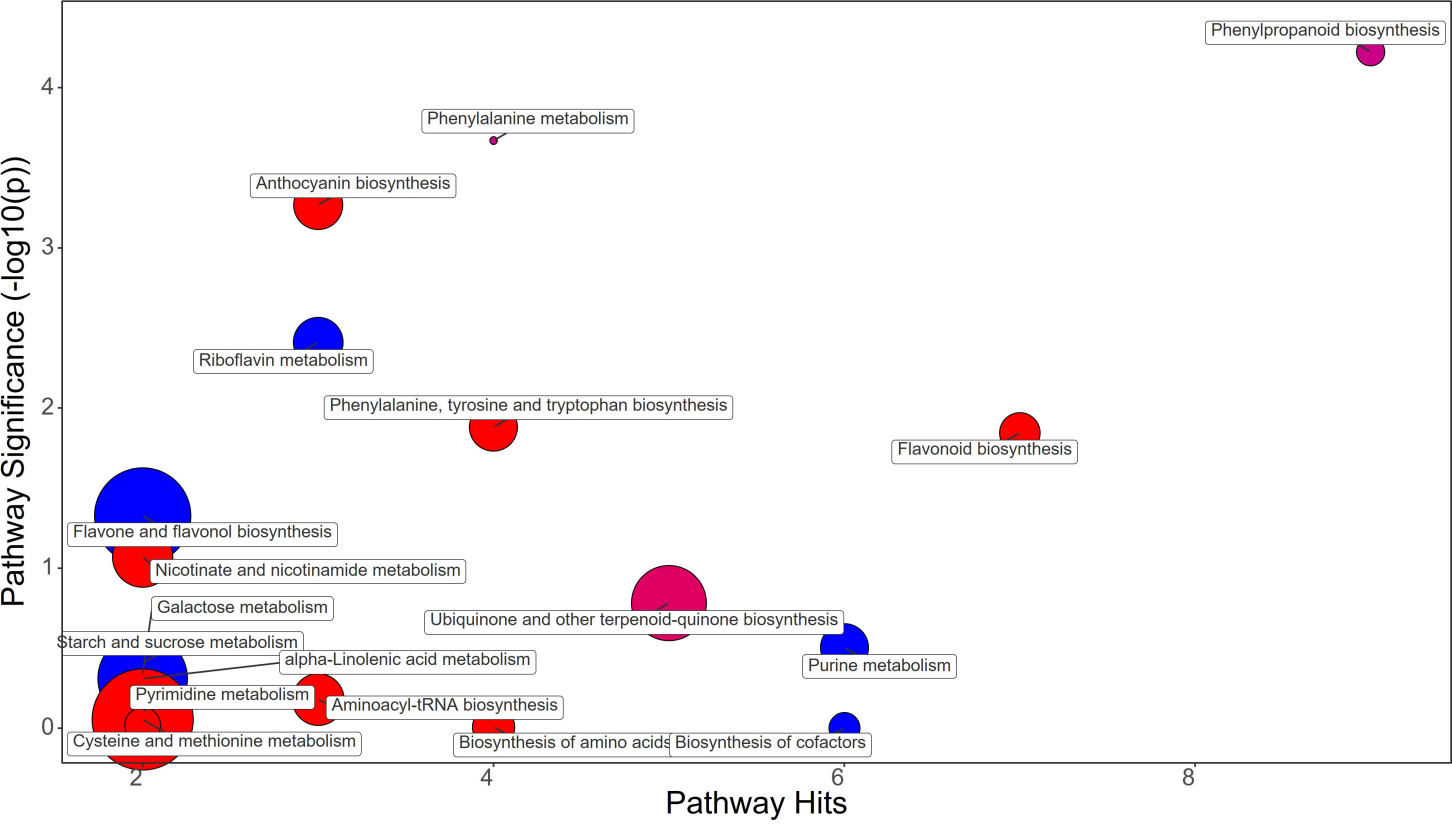
Chemical similarity enrichment analysis (ChemRICH) of statistically different annotated metabolites in synthetic auxins treated fruits compared to untreated control of strawberry plants. Pathway significance (-log10(p)) is plotted on the y-axis, while the pathway hits are represented on the x-axis. Clusters are color-coded to indicate the proportion of compounds that have increased (in red), decreased (in blue), or exhibited mixed changes (in purple).

**Figure 8:**
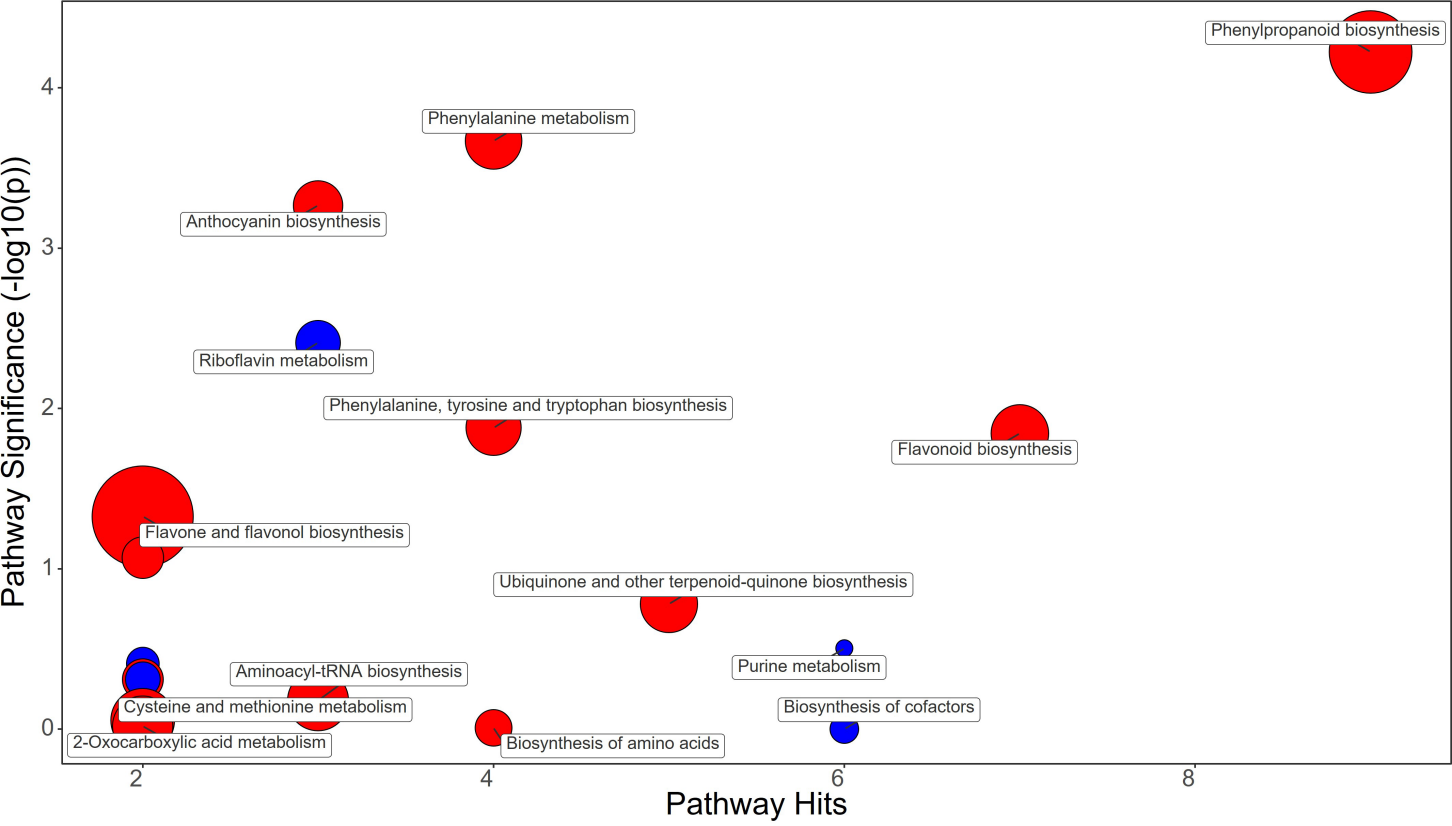
Chemical similarity enrichment analysis (ChemRICH) of statistically different annotated metabolites in protein hydrolysate treated fruits compared to untreated control of strawberry plants. Pathway significance (-log10(p)) is plotted on the y-axis, while the pathway hits are represented on the x-axis. Clusters are color-coded to indicate the proportion of compounds that have increased (in red), decreased (in blue), or exhibited mixed changes (in purple).

Metabolomic analysis revealed that strawberry fruits treated with BF, PH, and SA had distinct metabolic profiles (**Figures 6**–**8**). Despite this fact it has been highlighted that all treatments increased the flavonoid content in the fruits through different metabolic mechanisms.

Bacterial filtrate treatment did not alter Phenylalanine biosynthesis in fruits, but the phenylpropanoid pathway showed an increase in Cinnamaldehyde (FC=1.3) and Ferulate (FC=1.16) with a down-regulation of trans-Cinnamate (FC=0.7) (**Figure 6**; **Supplementary File 1**). Among the metabolites that also were up-regulated, Catechin (FC=1.26) and the anthocyanins Delphinidin (FC=1.21) and Delphinidin 3-O-glucoside (FC=1.12) were significantly increased by BF treatments (**Supplementary File 1**).

Synthetic auxins treatment did not alter Phenylalanine biosynthesis, and even the phenylpropanoid pathway showed a decrease in trans-Cinnamate (FC=0.54) in strawberry fruits (**Figure 7**; **Supplementary File 1**). However, an enhancement of Catechin (FC=1.51) and the anthocyanins Delphinidin (FC=1.46) and Pelargonidin 3-O-glucoside (FC=1.29) was recorded with the application of SA (**Supplementary File 1**).

Protein hydrolysate treatment significantly increased the content of Phenylalanine (fold change, FC=2.1), stimulating the phenylpropanoid pathway as evidenced by the increase in Ferulate (FC=1.4) and Coniferyl alcohol (FC=11.43) in strawberry fruits (**Figure 8**; **Supplementary File 1**). The flavonoids Kaempferol (FC=1.33) and Catechin (FC=1.33) were also increased in PH-treated fruits, as were the anthocyanins Delphinidin (FC=1.68), Delphinidin 3-O-glucoside (FC=1.21), and Pelargonidin 3-O-glucoside (FC=1.12) (**Supplementary File 1**).

## 4 Discussion

Results of the agronomic trial demonstrated that synthetic auxins (SA) and bacterial filtrate (BF) significantly enhanced early yield of strawberry fruits due to a positive effect of auxins on flowering and fruit set. Several researches reported the role of SA in inducing fruit set in many species (Srivastava, 2002; Stern et al., 2007; Sun and Hong, 2009; Yan et al., 2014) and in strawberry plants (Hunter, 1941; Thompson et al., 1969; Mudge et al., 1981) thus increasing the early fruit production. On the other hand, the protein hydrolysate (PH) favored a more gradual and prolonged fruit set over time with a greater development of the shoot biomass and total fruit number at the end of the growing cycle. This increased of shoot biomass, which is composed mainly of leaves (source tissue), could have augmented the photosynthetic capacity to sustain long-term production while the reduction in shoot biomass due to the great allocation of photosynthates on early fruit production (sink tissue) in auxin-treated plants (SA and BF) could have reduced the availability of photosynthates for new leaf development thus reducing the source activity (leaf photosynthesis) over time. Rylott and Smith (1990) also reported that application of synthetic auxin increased plant yield and made generative organs competitive over vegetative ones. It is well-known that PH ‘Trainer^®^’ bioactive compounds (peptides and amino acids) increase the plant’s photosynthetic activity, which in turn increases yields (Colla et al., 2015; Rouphael et al., 2017). Particularly, the typical components of tested vegetal-PH which act as signaling molecules, may have enhance accumulation of endogenous phytohormones like auxins, cytokinin’s and gibberellins (Colla et al., 2014; Ertani et al., 2017; Ceccarelli et al., 2021) to activate a signal transduction pathway, increasing crop yield (Rouphael and Colla, 2018 ). The prolonged total fruit production and plant biomass increase was found out similarly by some authors who observed that the use of two biostimulants derived from vegetal origin (alfalfa hydrolyzed and red grape skin), resulted in an increase in total fresh fruit weight and total fruit number along the whole greenhouse chili pepper crop cycle (Ertani et al., 2014).

The increase of crop yield in PH-treated plants was associated with an enhancement of total N concentration in leaves resulting from a positive effect of PH on the absorption of mineral nitrogen (Colla and Rouphael, 2015). Moreover, Sestili et al. (2018) showed an increase in N concentration of tomato leaves after the foliar application of the PH Trainer^®^ and referred this augmentation to the root growth stimulation and to the overexpression of genes implicated in N assimilation process. Selenium was also higher in leaves treated with PH respecting to SA treated and untreated leaves. Selenium is a crucial element for the scavenging and control of free radicals of plants and it is also an antioxidant, anti-senescent, abiotic stress modulator, and anti-senescent (Kaur et al., 2014). Therefore, the Se increase in strawberry leaves after PH foliar sprays may represent a positive attribute for maintaining an efficient photosynthetic apparatus over time. The decrease in leaf concentrations of B and Ni and trace elements such as Sb, TI, V, Co, As and Be in SA, and BF treatments can be related to a limited root activity resulting from the great allocation of photoassimilates to early fruit production whereas the decrease of leaf concentrations of B and Ni and trace elements (Sb, TI, V, Co, As and Be) in PH treatment can be attributed to the dilution effect induced by the increase of plant biomass resulting from Trainer^®^ applications. Leaf (1973) also reported that the levels of nonlimiting growth elements in plant tissues may decrease in concentration due to an increase of plant biomass.

Moreover, the results of mineral composition indicated that macronutrients in strawberry leaves of all treatments were within the following sufficiency ranges (Mills and Jones, 1996): N (21-40 g/kg), P (2.0-4.5 g/kg), K (110-250 g/kg), Ca (6-25 g/kg), and Mg (2.5-7.0 g/kg); leaf micronutrients such as Fe (50-250 mg/kg), Mn (30-350 mg/kg) and Zn (20-50 mg/kg) were also in the sufficient ranges reported by Mills and Jones (1996), whereas Cu (6-20 mg/kg), B (25-60 mg/kg) and Mo (0.25-0.50 mg/kg) were above the sufficient ranges in all treatments. The above findings indicated that nutrients were sufficient to maximize fruit yield in all treatments.

According to Ornelas-Paz et al. (2013), fruit quality characteristics including color, firmness, and chemical composition affect customer choice. Fruit firmness is a critical quality trait that affects the postharvest shelf life and marketability of fruits (Goulao and Oliveira, 2008). El-Sharkawy et al. (2016) reported that auxin play an indirect function in stimulating fruit ripening through the up-regulation of genes encoding for several ethylene components, leading in ethylene-induced fruit ripening and softening. This was not the case in our study since fruits treated with SA showed a similar firmness of untreated control.

Total soluble solids (TSS) of strawberries represent the amount of carbohydrates, organic acids, vitamins, amino acids, and pectin in fruit pulp (Ashour et al., 2023). This fruit quality trait plays an important role in determining the fruit taste beside the total acids and their ratios (Krüger et al., 2012). The reduction of TSS content in fruits treated with SA in comparison with control and PH may be related to the sink-source imbalance causing reduction of photosynthetic capacity and total soluble carbohydrate accumulation. The lower soluble solids content observed in SA-treated fruits suggests that synthetic auxins may have a negative effect on fruit sweetness (Suman et al., 2017), probably by altering sugar metabolism or transport (Gibson, 2004). Moreover, the analysis of fruit quality traits revealed significant differences between the SA and BF treatments on mineral concentrations of B, Al and Ti. Notably, the BF, a natural auxin, tended to outperform SA in maintaining fruit firmness and soluble solids content, two key indicators of fruit quality (Døving and Måge, 2001). Fruits treated with SA and PH showed a reduction in concentrations of B and some potentially harmful elements for human health such as Ni, Cd, Pb in comparison with the untreated fruits. Foliar treatments with BF induced a reduction in Pb and Cr in comparison to the untreated fruits. Titanium concentration was decreased in fruits after the applications of SA compared to the PH and BF treatments. Considering the dry matter of the fruits (**Table 3**), it is possible to express the concentrations of heavy metals such as Cd and Pb in fresh weight (f.wt.) basis as follow: 0.0028, 0.0038, 0.0028 and 0.0029 mg Cd/kg f.wt. for control, SA, BF and PH, respectively; 0.0093, 0.0018, 0.0047and 0.0022 mg Pb/kg f.wt. for control, SA, BF and PH, respectively. The above Cd and Pb concentrations in all treated and untread fruits were below the maximum permissible levels (0.050 mg Cd/kg f.wt.; 0.10 mg Pb/kg f.wt) reported in Regulations (EU) 2021/1317 and 2021/1323 for strawberry. The reduction of Cd and Pb accumulation in fruits under foliar sprays with SA, and BF could be associated to a reduction of root activity in SA and BF treatments resulting from the great allocation of photosynthetates in the fruits at the expense of shoot and root growth. Moreover, the highest fruit yield in PH-treated plants could have reduced the Cd and Pb accumulation in fruits by dilution effect.

Primary and secondary metabolism have been widely categorized as two stages of plant metabolism. The first step in the process of creating primary macromolecules such proteins, carbohydrates, nucleic acids, lipids, and hormones is the primary metabolism pathway which is responsible of supporting photosynthesis, plant growth and development (Bernards, 2010; Zhao et al., 2021). Secondary metabolites are derived from the primary metabolic pathways (Hussein and El-Anssary, 2019) and are composed of flavonoids, terpenoids, alkaloids, sterols, steroids, essential oils, lignin, carotenoids, polyphenols, anthocyanins that can act as antioxidant and defensive molecules against abiotic and biotic stress (Anjali et al., 2023). In this study, SA induced an accumulation of secondary metabolites thus reducing the carbon and sugar necessary for plant growth and production. Elmongy et al. (2020) reported that application of the synthetic auxin naphthaleneacetic acid promoted H_2_O_2_ production in azalea microshoots, possibly via increased peroxidase (POD) activity, because POD can participate in oxidative metabolism and in the production of H_2_O_2_; this may be the case in the current trial where SA promoted the accumulation of secondary metabolites such phenolic compounds for reducing the activity of reactive oxygen species like H_2_O_2_ in plant cells. Similar results to SA treatment on secondary metabolism were observed for BF treatments indicating that BF has the potential to replace SA for early production of strawberry fruits. On the other hand, PH promoted an accumulation of primary metabolites inducing a great support of photosynthesis and plant use of energy for plant growth over a longer period of the plant cycle resulting in a longer fruit production and higher yield. Several studies showed that the application of PH can boost crop yields and performance by activating carbon primary metabolism and N assimilation (Colla et al., 2017; Rouphael et al., 2018; Malécange et al., 2023).

A comparative metabolomic analysis was performed for discerning the metabolic pathways differentially affected by applications of PH, SA and BF. PH treatment resulted in an increase in Phenylalanine and Tyrosine, two amino acids that play crucial roles in plant growth and development. However, PH induced a decrease of Chorismate and Tryptophan, which are precursors of many plant growth regulators like auxin and salycilic acid (Erland and Saxena, 2019). This could have contributed to the delay in production observed in PH-treated plants. The decrease in Ferulate and Syringin in the phenylpropanoid pathway, along with a decrease in the anthocyanin Delphinidin 3-O-glucoside and Pelargonidin 3-O-glucoside, might have affected leaf coloration and overall plant health (Ramaroson et al., 2022). The PH-mediated effect on purine metabolism as indicated by the decreased levels of Guanine, dADP, Adenosine, and Deoxyadenosine, might have resulted from an enhancement of purine catabolism in plants which is a key process for recycling N in plant cells for remobilization to support new growth and reproduction (Zrenner et al., 2006). Moreover, several studies highlighted that purine degradation is also closely linked to plant responses and adaptation to stress. For example, several plants respond to environmental stress by inducing and activating enzymes in the purine degradation pathway (Watanabe et al., 2014). SA treatment also increased Phenylalanine, Phenylacetaldehyde, Tyrosine, Chorismate, and Tryptophan in leaf tissues. This suggests an overall stimulation of amino acid synthesis and phenylpropanoid pathway, which could have contributed to the early fruit production in SA-treated plants (Singh et al., 2010). The increase in Ferulate, Scopoletin, Coniferyl alcohol, and Coumaroyl quinic acid, along with an increase in the flavonoids Naringenin, Kaempferol, Gallocatechin, and Delphinidin, might have play a role as antioxidants in protecting plant cells from reactive oxygen species (ROS) generated by auxin application. BF treatment resulted in a similar metabolic profile to SA treatment, with an increase in Phenylalanine, Phenylacetaldehyde, Tyrosine, Chorismate, and Tryptophan. The increase in Ferulate, Scopoletin, Coniferyl alcohol, and Coumaroyl quinic acid, along with an increase in the flavonoids Catechin, Naringenin, Kaempferol, Gallocatechin, and Delphinidin, suggests that BF may also have enhanced antioxidant activity of plant cells for alleviating ROS damage.

The metabolomic analysis of strawberry fruits treated with BF, SA, and PH highlighted contrasting metabolic profiles, which were reflected in the observed differences in fruit quality. PH treatment led to an increase in Phenylalanine content of fruits, a precursor for many phenylpropanoids, and stimulated the phenylpropanoid pathway, leading to an increase in beneficial metabolites such as ferulate and coniferyl alcohol. This was accompanied by an increase in the flavonoids Kaempferol and Catechin, and the anthocyanins Delphinidin, Delphinidin 3-O-glucoside, and Pelargonidin 3-O-glucoside. These metabolites are known to contribute to fruit quality by enhancing color, flavor, and nutritional value (Parra-Palma et al., 2020). It is well known that a consumption of anthocyanins and flavonoids-rich fruits and vegetables contribute positively to human health mitigating cardiovascular disease, type 2 diabetes, non-alcoholic fatty liver disease, and neurological disorders (Kozłowska and Szostak-Węgierek, 2018; Oteizaìa et al., 2023 ). The increase in these metabolites suggests that PH treatment may enhance the fruit quality by stimulating the phenylpropanoid pathway and increasing the production of beneficial flavonoids and anthocyanins. SA treatment did not affect Phenylalanine biosynthesis and resulted in a decrease in trans-Cinnamate in the phenylpropanoid pathway. However, there was an increase in Catechin and the anthocyanins Delphinidin and Pelargonidin 3-O-glucoside. This suggests that SA treatment may enhance fruit quality through different metabolic mechanisms, possibly by directly stimulating the biosynthesis of beneficial flavonoids and anthocyanins. BF treatment resulted in a similar metabolic profile of SA treatment, with an increase in Cinnamaldehyde and Ferulate, and a decrease in trans-Cinnamate in the phenylpropanoid pathway. This was accompanied by an increase in Catechin and the anthocyanins Delphinidin and Delphinidin 3-O-glucoside. This suggests that BF treatment may also enhance fruit quality through similar metabolic mechanisms of SA, possibly by stimulating the biosynthesis of beneficial flavonoids and anthocyanins.

## 5 Conclusion

The results of the agronomic trial offer valuable insights into the effects of synthetic auxins (SA), bacterial filtrate (BF), and vegetal-derived protein hydrolysate (PH) on strawberry fruit production and quality. The foliar applications of SA and BF significantly boosted early fruit yields, primarily attributed to their positive impact on flowering and fruit set. In contrast, PH treatment favored a gradual and prolonged fruit set, leading to increased shoot biomass and total fruit numbers over the entire growing cycle. This sustained production was likely due to the enhanced photosynthetic capacity supported by PH’s bioactive compounds, such as peptides and amino acids, which are expected to activate key phytohormones. Our study also revealed differences in fruit quality among the treatments. PH-treated fruits exhibited improved firmness and soluble solids content if compared with SA treatment, but no significant differences were observed in comparison with control; fruit firmness and soluble solids contents are essential factors in determining postharvest shelf life and consumer preference. On the other hand, SA-treated fruits displayed lower firmness and soluble solids content, raising concerns about the effects of SA on fruit sweetness. Furthermore, the analysis of nutrient concentrations in leaves and fruits demonstrated that all treatments provided sufficient macronutrients for maximizing fruit yield and remained within regulatory limits for potentially harmful elements. PH treatment showed the most promise in reducing the accumulation of heavy metals in fruits, if referred to dry mass. Metabolomics indicated that the PH treatment stimulated primary metabolites, enhancing photosynthesis, and supporting long-term growth, while SA and BF treatment directly affected the biosynthesis of beneficial flavonoids and anthocyanins, contributing to enhanced fruit quality. In conclusion, this study highlights that SA may expedite early fruit production but might affect fruit firmness and sweetness, while PH treatment prolongs fruit set and supports photosynthetic capacity, leading to sustained production and improved fruit quality. BF treatment, with its natural auxin content, is a viable option to SA for enhancing fruit firmness and potentially influencing flavonoid and anthocyanin biosynthesis. Understanding the unique effects of these treatments provides valuable insights for growers and researchers seeking to optimize strawberry production and fruit quality.

## Data availability statement

The original contributions presented in the study are included in the article/**Supplementary Material**. Further inquiries can be directed to the corresponding authors.

## Author contributions

MCa: Conceptualization, Data curation, Formal analysis, Investigation, Methodology, Software, Supervision, Validation, Writing – original draft, Writing – review & editing. AE: Data curation, Formal analysis, Methodology, Validation, Writing – original draft, Writing – review & editing. YR: Methodology, Software, Writing – review & editing. MCi: Software, Writing – review & editing. PB: Conceptualization, Data curation, Formal analysis, Funding acquisition, Investigation, Methodology, Project administration, Resources, Software, Supervision, Validation, Visualization, Writing – original draft, Writing – review & editing. GE: Writing – review & editing. VC: Resources, Visualization, Writing – review & editing. BB: Data curation, Methodology, Software, Writing – review & editing. GCor: Methodology, Software, Writing – review & editing. SC: Methodology, Writing – review & editing. H-JK: Methodology, Writing – review & editing. GCol: Conceptualization, Data curation, Formal Analysis, Funding acquisition, Investigation, Methodology, Project administration, Resources, Software, Supervision, Validation, Visualization, Writing – original draft, Writing – review & editing.
